# Addressing Health Literacy by School Nurses at Schools Across Germany: Results of a Mixed‐Methods Study

**DOI:** 10.1111/jocn.70472

**Published:** 2026-07-31

**Authors:** Jana Kaden, Lia Schilling, Birte Berger‐Höger

**Affiliations:** ^1^ Department Evaluation and Implementation Research in Nursing Science University of Bremen, Institute for Public Health und Nursing Research Bremen Germany

**Keywords:** empowerment, health education, health literacy, health promotion, school, school nursing

## Abstract

**Background:**

Health literacy has become increasingly important in healthcare and education. The aim is to provide an overview of school nurses' activities to increase health literacy in schools, and to analyse their conceptual understanding of health literacy.

**Methods:**

This mixed‐methods study consists of an online survey (*n* = 65), two focus group interviews (*n* = 16) and non‐participatory observations with school nurses in Germany (*n* = 5), and expert interviews with interest‐holders (*n* = 10). School nurses' health literacy‐related activities, conceptual health literacy understanding, and critical health literacy (measured by the Critical Health Competence Test) were assessed. Quantitative data were analysed descriptively, and the qualitative data by content analysis. Quantitative and qualitative findings were integrated and triangulated using a convergent design.

**Results:**

School nurses address health literacy among students and their relatives through counselling, teaching support, and acute care. Their services are primarily based on a functional health literacy understanding. Experts view schools as key settings for health literacy, led by school nurses.

**Implications for the Profession and/or Patient Care:**

School nurses use real‐life oriented, low‐threshold activities to address health literacy and empower students. School nurses' critical health literacy and evidence‐based practice could be improved.

**Conclusions:**

School nurses represent a valuable resource to address health literacy in German schools, in line with international approaches. These interventions should be expanded.

**Reporting Method:**

This study followed the criteria for Good Reporting of A Mixed Methods Study (GRAMMS), the consolidated criteria for reporting qualitative research (COREQ) and the Checklist for Reporting Results of Internet E‐Surveys (CHERRIES).

**Patient or Public Contribution:**

No direct patient or public contribution.

## What Does This Paper Contribute to the Wider Global Clinical Community?

- This paper highlights the potential of school nurses as key actors in promoting health literacy among students and their environments.
- It suggests expanding existing interventions beyond a functional understanding of health literacy toward a more comprehensive approach that integrates critical health literacy to empower students.
- It provides opportunities for reflection and further development of health literacy interventions, especially in school settings.

**Abbreviations**
CHERRIES: Checklist for Reporting Results of Internet E‐Surveys
COREQ: Consolidated Criteria for Reporting Qualitative Research
DBfK: German Nursing Association
GRAMMS: Good Reporting of A Mixed Methods Study
WHO: World Health Organization

## 1 Background

Health literacy is a determinant of health and has gained importance within healthcare and education sector (BMG 2024; Sørensen et al. 2012). It is a complex concept which includes the ability to find, evaluate and apply health information (Sørensen et al. 2012). Functional, interactive, and critical health literacy are well‐established dimensions (Nutbeam 2000; Sørensen et al. 2012). Critical health literacy, in particular, empowers individuals to make informed health decisions and take actions for their health and well‐being within their own social and cultural context (de Wit et al. 2018). Many people in Germany and other European countries experience challenges related to health literacy (Baccolini et al. 2021; Schaeffer et al. 2021). Following the ‘Integrated model of health literacy’, health literacy encompasses the domains healthcare, disease prevention, and health promotion, and is acquired through a lifelong learning process at both, the individual and population level (Sørensen et al. 2012). Designing setting‐based interventions to increase health literacy is recommended (BMG 2024; WHO 2017). Schools are key settings for the early, long‐term and longitudinal development of (critical) health literacy among children and adolescents (Nash et al. 2021; Paakkari et al. 2019). Given the complexity of developing critical health literacy, it should be embedded as a cross‐cutting theme across different subjects (Bruselius‐Jensen et al. 2017; Nash et al. 2021). Nevertheless, teaching staff often lack confidence in teaching or integrating health and health literacy (Nash et al. 2021). School nurses are gaining importance as potential multipliers of health‐related knowledge and skills. Nurses enhance health literacy according the updated nursing definition (White et al. 2025). While in different countries school nurses are already integrated in schools and addressing health literacy is part of their tasks (Kaden and Berger‐Höger 2025b; Pawils et al. 2023; WHO 2021), the implementation in Germany is still in an early stage (Ellsäßer and Manz 2025; Kaden and Berger‐Höger 2025a). Germany lacks uniform standards or legal regulations for school nurses (Kaden and Berger‐Höger 2025a). This leads to diverse school nursing approaches and the predominantly non‐academic school nurse qualification (In Germany, professional qualification as a nurse can be obtained through two pathways: a 3‐year vocational training programme below bachelor's level or a bachelor's degree programme.) in line with the general nursing qualification (Kaden and Berger‐Höger 2025a). Initial findings from pilot projects in Germany suggest a potential benefit of school nurses on students' health literacy and their environment (de Buhr et al. 2020).

Despite growing interest in school‐based health promotion strategies, little is currently known about specific measures to address health literacy in schools in Germany, and the related school nurse's role.

### 1.1 Aim

The presented study aims to provide an overview of activities to enhance health literacy in German schools in which school nurses are involved, to examine which dimensions of health literacy are prioritised, and to explore school nurses' understanding—particularly of critical health literacy. Findings aim to inform the scientific discussion in advancing school‐based health literacy programmes and guide the future development of the school nurse's role.

This leads to the following research questions:

- What activities to increase health literacy are offered by school nurses?
- Which type of health literacy is addressed by the school nurses' activities?
- What health literacy understanding and level of critical health literacy do school nurses have?

Additionally, the perspectives of relevant interest‐holders regarding health literacy in schools and the school nurses' role are assessed.

## 2 Methods

This study was part of an exploratory survey and context analysis of school nursing in Germany (Kaden and Berger‐Höger 2025a). The study protocol was published in advance (Kaden and Berger‐Höger 2022). We used a convergent mixed‐methods study design (Creswell and Plano Clark 2018). The results of the quantitative survey were compared, validated, and supplemented with the findings of the qualitative investigations in order to contextualise and explain them, and to generate a deeper understanding (Halcomb 2019).

First, an anonymous open online survey (S) among school nurses in Germany was conducted. Afterwards, semi‐structured, guided focus group interviews (FG) and non‐participatory observations with school nurses (observation, O) and expert interviews (E) (Bogner et al. 2014) with relevant interest‐holders were conducted.

This study has been reported in accordance with the consolidated criteria for reporting qualitative research (COREQ) (Tong et al. 2007) (Appendix S1), the Checklist for Reporting Results of Internet E‐Surveys (CHERRIES) (Eysenbach 2012) (Appendix S2) and guided by the criteria for Good Reporting of A Mixed Methods Study (GRAMMS) (O'Cathain et al. 2008).

### 2.1 Participants

School nurses who have been active in Germany within the past 5 years were eligible to participate in the *survey*. Based on data from the school nursing network of the German Nurses Association (DBfK), 80 school nurses were estimated to be eligible for the study. The aim was to achieve the highest possible response rate to represent the field range. At least one *focus group interview* and five *non‐participatory observations* with school nurses were planned. To ensure diversity, school nurses from different federal states, representing different conceptual approaches were included. Within focus groups, participant homogeneity was prioritised (e.g., same federal state). At least five *expert interviews* with relevant interest holders linked to the role of school nurses were planned.

School nurses were recruited by using snowball sampling. Invitations were sent via email to employers and the DBfK's network coordination, with a request for forwarding. A reminder was subsequently sent. Additionally, following completion of the online survey, interested school nurses could leave their contact for qualitative data collection. Expert interview participants were also recruited via snowball sampling, using personal contacts, networks, conferences and the observations.

### 2.2 Data Collection

Next to the critical health competences test (CHC‐test) (Steckelberg et al. 2009), no validated questionnaires for the different data collections of the study were identified. Therefore the assessment forms were developed literature based, using the four‐step method (Helfferich 2014), and refined accordingly with experts' feedback (practice and university setting).

### 2.3 Survey

Closed survey questions were used to assess to what extent school nurses address health literacy. Open‐ended questions were used to explore school nurses' subjective health literacy understanding and activities related to health literacy at school and the school nurses' involvement. Additionally, sociodemographic data were collected. The school nurses' critical health literacy was objectively measured using the critical health competences test (CHC‐test), a valid and reliable instrument (Steckelberg et al. 2009). It comprises four different scenarios, each represented in four test sections containing a total of 72 items. The first section consists of four different and relevant content areas of healthcare. The second section is built up with four subareas of competence, representing the underpinning theoretical structure of the critical health literacy construct. These categories are: A – understanding medical concepts; B – skills of searching literature; C – Basic statistics; and D – design of experiments and sampling (Lühnen et al. 2020; Steckelberg et al. 2009). The instrument demonstrated strong construct validity (Cohen's *d* = 4.33, 95% CI [3.51, 5.16]) and test reliability according to the Rasch model (ANOVA) is 0.91, and the reliabilities for the individual scenarios are 0.71 (Scenario 1), 0.78 (Scenario 2), 0.75 (Scenario 3) and 0.80 (Scenario 4) (Steckelberg et al. 2009). The Rasch scaled person parameters range from 0 to 1000, with higher values indicating greater critical health literacy (Steckelberg et al. 2009). For the present study, Scenario 1 of the CHC‐test, consisting of 17 items, was selected.

Completing the overall online survey took approximately 30–45 min. It was conducted via LimeSurvey (2022). The mandatory question on school nurses' employment status served as a filter for inclusion. Two persons pre‐tested the survey. The survey was open from December 2022 to February 2023.

### 2.4 Qualitative Investigations

The guiding research questions for focus groups and observations were: From the perspective of school nurses, what potential and interventions are there to enhance students' health literacy in schools? To what extent is addressing (critical) health literacy part of the role profile of school nurses in Germany?

If participants were unfamiliar with the concept of health literacy, it was explained by the researcher.

Focus groups with additional field notes were conducted either in person on‐site or digitally via Zoom (JK, LS). During non‐participatory observations, the researcher (JK) accompanied participants for one full school day and recorded field notes. A joint discussion followed to contextualise, validate, and explore the findings. Sociodemographic data to describe the sample were collected paper‐based or via LimeSurvey (2022).

Within the expert interviews, perspectives of interest‐holders on addressing critical health literacy within schools, the context of school nursing and necessary competences in Germany were explored. Their professional qualifications, activities, and relationship to school nursing were collected as sociodemographic data. The interviews were conducted according to participants' preferences either by telephone, online via Zoom, or in person at the researcher's workplace.

The qualitative data collection took place between February and June 2023 until data saturation was reached. Data saturation was reached once enough information had been collected to answer the research questions and achieve the research objectives. This was discussed by the research team throughout the research process. All interviews were digitally recorded, transcribed verbatim, and anonymised.

### 2.5 Data Analysis

The quantitative data (JK) were analysed descriptively using SPSS, version 28 (IBM Corp 2021). Data of the CHC‐test were previously reviewed by a second researcher (JK, AMP).

All qualitative data (open‐ended survey responses, interviews and field notes) were analysed together, using content analysis oriented on Mayring (2014) with the software MAXQDA 2022/2024 (VERBI Software 2023) and independently reviewed by a second researcher (JK, LS). A deductive coding scheme based on the interview guides was developed and supplemented by inductively identified categories during analysis. To analyse the school nurses' understanding of health literacy, deductive categories were derived from theoretical definitions: functional, interactive/communicative and critical health literacy were oriented on Nutbeam (2000), Sørensen et al. (2012) and Benkert and Abel (2020). Professional health literacy was based on Schaeffer et al. (2023) and digital health literacy on Kolpatzik et al. (2020). Physical activity and nutrition were guided by Jordan et al. (2025).

The anonymised results of one focus group underwent communicative validation (Flick 2018) with two participant‐representatives; they confirmed the results. All analysed data were combined, compared, and iteratively discussed within the research team.

### 2.6 Ethical Considerations

The ethical approval for the study was provided by the ethics commission of the University of Bremen (file number: 2022‐17). Participation in the study was voluntary and unpaid. All potential participants received written study information and informed consent was obtained prior to data collection. For the observations, additional consent from the employer or school leader was required.

## 3 Results

A total of *n* = 65 datasets from school nurses were included in the analysis of the online survey. As incomplete questionnaires were included, the respective sample size (*n*‐value) is reported. Two focus group interviews were conducted (54 and 74 min) with a total of 16 school nurses. Of six interested school nurses, five obtained formal approval from their employer and were observed for one full workday (5.5–8 h). Additionally, 10 experts were interviewed (mean duration 42 min, range 18–71 min).

The results are reported by the following aspects: addressing health literacy with details on activities of school nurses and addressing critical health literacy and materials used in practice; the expert's perspective on addressing health literacy by school nurses as well as critical health literacy and evidence‐based practice; the results of the school nurses' understanding of health literacy and the results of the CHC‐test. For qualitative categories and their respective subcategories with example codes see Appendix S3.

### 3.1 Participants' Sociodemographic Data

School nurses from different federal states in Germany participated in the respective data collections. An overview of the sociodemographic data is presented in Table 1. The participating school nurses were employed in different school types; most work in primary schools (grades 1–4 or 1–6, federal state‐dependent). School nurses from international schools were only represented in the online survey. The professional qualification of most school nurses is as a nurse or paediatric nurse, which is a three‐year vocational training. Additionally, *n* = 22 of the online survey participants and 12 of them in the focus group interviews held a Bachelor's degree in nursing science, public health or a related field. Most school nurses were in the age groups of 25 years and older, and the professional experience ranged from 1 year to more than 10 years, except some within the online survey were employed as a school nurse for less than 1 year.

**TABLE 1:** Sociodemographic data of the participating school nurses.

Number of different federal states	Online‐Survey	Focus Groups 1 and 2^a^	Observations^a^
	*n* = 64	*n* = 16	*n* = 5
	12	4	4
City size—inhabitants	**61** ^d^	**16**	**5**
> 500,000	25	11	4
100,000 to 500,000	16	2	—
50,000 to < 100,000	5	—	1
20,000 to < 50,000	6	3	—
10,000 to < 20,000	6	—	—
5000 to < 10,000	3	—	—
Age group	**63**	**16**	**5**
< 25 years	1	—	—
25–35 years	23	8	1
36–45 years	12	2	1
46–55 years	18	3	1
> 55 years	9	3	2
Gender	**63**	**16**	**5**
Female	62	15	5
Male	—	1	—
I do not want to categorise myself by gender	1		
Professional background (multiple answers)	**63**	**16**	**5**
Geriatric nurse	1	—	—
Paediatric nurse	26	4	3
Nurse	33	12	2
Bachelor's degree^b^	22	12	2
Specialist training: school health professional	10	3	1
Other^c^	22	6	2
Professional experience as school nurse	**64**	**16**	**5**
< 1 year	18	6	—
1–5 years	31	6	2
6–10 years	6	3	2
> 10 years	9	1	1
School type (multiple answers)	**64**	**16**	**5**
Primary school	44	15	4
International school	10	—	—
School kindergarten/pre‐school	8	1	—
Gymnasium/grammar school	8	1	—
Integrated comprehensive school	5	2	1
School with several educational programmes	4	1	1
Secondary school	4		
Special school	3	1	—
Other types of school	2		
Number of assigned schools	**62**	**16**	**5**
1	45	8	4
2	15	7	1
More than 2	2	1	—
Number of students	**59**	**16**	**5**
< 50	3		—
50–299	6	3	2
300–499	18	5	2
500–999	21	6	1
1000–1499	7	2	—
≥ 1500	4		—

^a^ Due to anonymization requirements, participants sociodemographic data are reported in aggregated form.

^b^ Bachelor's degree in nursing science, public health or a related field.

^c^ Master in nursing science/public health, Anaesthesia and intensive care specialist, practice instructor.

^d^ Respective *n* in bold.

The interviewed experts comprised three teachers, two project partners from projects on school nursing implementation, one school administrator, one educational specialist, one school social worker, one scientist, and one school nurse from Norway who participated.

### 3.2 Addressing Health Literacy

Overall, some school nurses described increasing students' health literacy as a key objective of their work (S, FG, O).

85% (*n* = 33) of the 39 school nurses within the online survey (multiple answers) are involved in strengthening health resources and health literacy. Each 92% (*n* = 36, each) offer counselling and health promotion, and situational and behavioural prevention within the school setting.

### 3.3 Detailed Activities of the School Nurse to Increase Health Literacy

Overall, the school nurses' involvement in school‐based activities to increase health literacy is different, and they adapt their offerings to the respective school conditions, the students' needs, and own individual strengths (FG, O, S). School nurses consider it important to continuously address health literacy.

In detail, school nurses' activities include *teaching and project work* on health‐related topics, partly also break activities. The topics include nutrition, first aid, addiction prevention, vital signs, eating disorders and diabetes, hygiene, physical activity, media consumption, and stress reduction. The extent of school nurses' involvement varies from the support of teaching staff in planning health‐related lessons and co‐teaching to independent teaching. Most school nurses describe an appreciative working relationship with the teachers. Many school nurses reported the requirement of a teacher's presence in the classroom during their activities. Sometimes the project work is in cooperation with external providers.

School nurses also address health literacy through *counselling and training* of students, their relatives and the school environment. These counselling services include both individual consultations and group training sessions, for example, as part of parents' evenings. Further services include ‘parent cafés’ or healthy breakfasts, offered jointly by the school nurse, teaching staff and school social workers.

Another activity is the *development of the school environment*. This includes sports programmes, healthy school food, the provision of drinking water (dispensers), a power room and logopaedic services. In addition, various opportunities for student participation, including surveys on school meals or the establishment of a student council, are provided.

Beyond that, school nurses collaborate in *health/project groups* at school. They develop concepts on topics such as bullying, sexual violence, addiction prevention, mental health, and health promotion at school. School nurses reported organising, developing content, supporting, or initiating these groups.

In some approaches, *acute care* is a task of school nurses which includes the provision of information about health‐related issues, preventive measures and potential treatment options:

During treatment, I provide information about the causes and explain ways to alleviate the symptoms; show ways of prevention […] I explain how long it will take to recover. (S 82: 9)

Further initiatives reported include the ‘school first aid service’ with regular trainings led by the school nurses and to check vaccination record.

Some schools have not yet established programmes to address students' health literacy from the school nurses' perspective. Reasons reported are a related gap in the curriculum and/or lack of teaching staff with experience in health. A few school nurses reported not being involved in activities to increase health literacy. In these cases, the tasks are carried out by teachers and/or external organisations. Reasons given for the non‐participation of school nurses are the ongoing onboarding process or acceptance problems from teachers:

“Reluctantly, as teachers do not like to reveal their cards. Those who have not studied cannot impart health knowledge.”(S 46: 14)

### 3.4 Addressing Critical Health Literacy by School Nurses

Critical health literacy and evidence‐based practice are currently addressed to some extent by school nurses. School nurses were open to critical health literacy and regard it as highly relevant to their professional practice. From their perspective, it should be addressed among students and their relatives, with the aim of empowerment. Examples of addressing critical health literacy in schools include information seeking and appraisal (with a focus on media use), reflecting critically about advertising, HPV vaccination and nutrition. School nurses expressed interest in expanding or integrating critical health literacy into existing programmes. Some school nurses suggest dealing with the topic from grade 3, compared to others, who perceive it as challenging due to its complexity and express concerns regarding students' varying reading/writing skills and attention spans.

### 3.5 Materials

School nurses seek appropriate materials that are tailored to the students' needs. Sometimes, they create their own materials to address topics continuously over a longer time period, or in the absence of suitable materials (FG, O).

So, there are a few materials I regularly use, or where I already know that good materials exist. But I also check that there aren't materials with so much text that they immediately end up in the corner and are never seen again. And I try to consider who my target group is. How old are the students? What are they like at the moment? What can I use, what can't I use? […] Because even if I provide really great, well‐prepared material, it ends up in the recycling bin and then I still haven't achieved anything. (FG2: 205–209)

School nurses consider high‐quality materials important. This is associated with trustworthiness, which is considered to be achieved due to its publication by official institutions or health authorities. Evidence‐based content was less frequently referred to as a quality criterion and for some the integration of social aspects is important. Besides that, the campaign character of materials with selectively presented information is partly reflected. Some school nurses have requirements from employers or cooperating physicians regarding the materials used. That includes the consistent use of materials across the team or even if the content is not always evidence‐based, it should at least be free from commercial interests. Some school nurses provide information materials publicly for students, while others use them targeted, for example, during counselling sessions.

### 3.6 Health Literacy at School—Experts' Perspective

The experts consider schools as central learning environment for addressing health. Health literacy is described as life‐relevant competence which is currently underrepresented in school practice and an important task for school nurses. From an expert perspective, school nurses are well positioned to address health literacy across age groups through classroom instruction, structured health education programmes, and individual counselling. Unlike teachers, they bring professional expertise, direct access to students and their school environment, and operate without the formal assessment of student performance.

“[Our school nurse] of course she has a solid background in this field, which we [teachers] don't have. And we have to be very broadly based […] when it comes to health, it's important to know […] exactly why and in what way this is important.”(E 4: 83–85).

### 3.7 Critical Health Literacy and Evidence‐Based Practice

From the experts' experience (scientist, international school nurse), activities to increase critical health literacy are offered by school nurses internationally. For example, in Norway, school nurses address it during lessons, partly with teachers. Health information is provided by the government, and school nurses have competencies and access to search in specific databases.

Evidence‐based professional practice by school nurses in Germany has not yet been fully established due to partly non‐academic nursing qualification. Skills in this area could be improved from the expert's view (project partners, scientist).

I think we might have to abandon the idea that this is about more physical exercise and healthy eating, yes. The range is much, much broader. And that's why [school nurses] need this expertise [critical health literacy]. I'm just not sure if all of them in Germany have it, internationally I would say yes, they all do Bachelor's degrees, so they will have basic scientific skills there as well. (E 5: 59)

Experts perceive a training intervention, to enhance critical health literacy among school nurses as valuable. This could provide meaningful support for their professional practice, and counselling or co‐teaching offerings. School nurses are perceived as the only health experts in schools, regularly confronted with new information and clinical conditions, which requires appropriate skills and competencies. Overall, the experts expressed a positive attitude towards school nursing.

### 3.8 School Nurses' Health Literacy Understanding

Content analysis results (FG, O, S *n* = 31) show a diverse understanding of health literacy by school nurses, example codes see Table 2. Some of the descriptions include a complex health literacy understanding, which means in addition to knowledge about health‐related information the ability to critically appraise and apply them independently.

**TABLE 2:** School nurses' health literacy understanding – example quotes.

Functional health literacy
– ‘managing one's own health/illness based on information’ (S 85: 7). – ‘For me, health literacy is the ability of a person to understand information/research/facts/advice on physical, mental, and emotional health and to use this information to act in their own best interests.’ (S 80: 8).
Critical health literacy
– ‘They're also supposed to learn, where they can find trustworthy information and how to evaluate them.’ (S 26: 10) – Being able to obtain information independently, to reflect on whether the sources used are good sources, are the sources evidence‐based, representative, or instead without references or untrustworthy. Acquiring the correct, updated, evidence‐based information. Critical also means being able to question things and to not believe everything that is presented […] children need to understand this, as well as the advertising impact on children or those who might not reflect and think about it […] and they should apply what they know in their learning and their actions. (O 2: 37)
Professional health literacy
– ‘Providing health information and services to people easy and understandable. Strengthen health resources in order to influence health‐related actions and behaviours.’ (S 40: 6)
Health literacy – Comprehensive understanding
– ‘Enabling children to make independent decisions about their health’ (S 22: 6)
Different understanding
– ‘Being able to connect nutrition/physical activity and so on and health.’ (S 76: 7)
– ‘paying attention to signals from the body, such as pain, rashes, fast heartbeat, etc. ‐ having a balanced diet—integrating exercise into daily life—knowing one's limits, […] identifying and being aware of one's own needs (sleep, hunger, thirst, resting)’ (S 15: 7)

Most descriptions of health literacy refer to *functional health literacy*, the ability to find, understand and apply health information. The perception of one's own body in terms of health knowledge, self‐perception and the ability to recognise and change unhealthy lifestyles was also frequently mentioned. Less frequently the ability to recognise health‐promoting or ‐inhibiting conditions and to utilise available support services. A few comments could be linked to *critical health literacy*. *Professional health literacy* is closely linked to the school nurses' professional role and was identified in three descriptions from the survey. A focus is on the transmission of knowledge to others, particularly in the sense of functional health literacy, for example by sharing health‐related information (e.g., on healthy lifestyles), sensitising students to health topics and promoting health knowledge with the goal of enabling its application in everyday life. No statements were assigned to the categories ‘digital health literacy’ and ‘communicative/interactive health literacy’.

Some school nurses' health literacy understanding diverges from established theoretical models (e.g., functional, interactive, and critical health literacy). Rather than considering health literacy as an overarching competence related to dealing with health information, they include specific thematic domains, such as nutrition, physical activity, and physical and mental health. This reflects a context‐specific, practice‐oriented perspective, focusing in particular on health‐promoting and ‐conscious behaviour to maintain physical and mental well‐being.

### 3.9 CHC‐Test

In the analysis of the CHC‐test (Steckelberg et al. 2009), *n* = 20 datasets from the online survey could be included. The calculated person parameters are averaged *M* = 382 (SD = 152, min. 71; max. 565). As higher values indicate greater critical health literacy, these results suggest that critical health literacy among school nurses in Germany is improvable.

## 4 Discussion

Overall, the activities of school nurses in implemented regions across Germany align with international developments in addressing health literacy of students and their social environment by school nurses (Kaden and Berger‐Höger 2025b; WHO 2021). School nurses' real‐life oriented, low‐threshold counselling and health education offerings for students and their environment are recognised as an effective strategy, contributing to empowerment (Dantas et al. 2025; WHO 2017). School nurses have the flexibility to shape the thematic focus, act partly autonomously, and engage closely with teaching staff. Through collaboration, such as team teaching, further training of school staff can be facilitated, which supports the development and maintenance of health literacy (Nash et al. 2021).

By addressing health literacy within acute care, next to health promotion and disease prevention, all three domains of health literacy (Sørensen et al. 2012) can be addressed. This allows health literacy interventions to be less fragmented and more comprehensive within the whole‐school setting (Nash et al. 2021). Depending on the school nursing model or approach (Kaden and Berger‐Höger 2025a, 2025b), the scope of strategies and measures to improve health literacy can be extended to the community setting. Furthermore, school‐based approaches may generate feedback effects through students' learning to their relatives (Nash et al. 2021).

Data from the 2025 Children's Health Report in Germany show that children and adolescents want to participate in health‐related decisions which are currently inadequately implemented (Koletzko et al. 2025). It is therefore important to foster critical health literacy at an early stage, enabling children and adolescents to develop competencies necessary to actively participate. This is in line with recommendations to expand school‐based programmes on health literacy (Bruselius‐Jensen et al. 2017; Nash et al. 2021).

Compared to countries such as Canada or Finland, health is not a formal subject in schools and health literacy is not explicitly integrated into curricula in Germany (JCSH 2008; 2016; Schulenkorf et al. 2021; Simovska et al. 2016). The surveyed teachers identified opportunities to integrate health topics within existing school subjects, demonstrating the feasibility.

To address reported implementation barriers of health literacy programmes by school nurses, such as limited acceptance of their qualifications and skills by school staff, an academic qualification for school nurses is recommended (Broussard 2007).

Overall, the findings suggest that participating school nurses lack a shared, coherent understanding of health literacy. Moreover, their understanding of it overlaps with the concept of health promotion and physical activity or nutrition interventions. This underlines the need to clearly distinguish between the concepts of health literacy and health promotion (Pawils et al. 2023), which operate on different conceptual levels (Sørensen et al. 2012). The proliferation of definitions and new terms in health literacy, along with inconsistent usage, risks semantically undermining the internationally established concept. Regarding critical health literacy, there is no uniform understanding within the scientific discourse (Abel and Benkert 2022; Benkert and Abel 2020). Inconsistent understanding and use of terms hinder the achievement, evaluation, and measurement of intervention objectives. An established, common understanding would enable school nurses to reflect on and improve existing interventions to enhance health literacy.

The results of the CHC‐test are comparable to the results of critical health literacy among nurses in Germany using the CHC‐test in other surveys (Hecht et al. 2021). Despite being comparable to results of a survey on the professional health literacy of nurses in Germany, even this is limited by using a self‐assessment instrument (Schaeffer et al. 2023). In summary, critical health literacy, including competencies in scientific methods and evidence‐based practice, is important for school nurses. They need to search and appraise material independently and provide information which could be supported by the provision of high‐quality, evidence‐based material. These findings are in line with international research on school nurses' information needs and necessary research skills required for their daily practice (Nickum et al. 2023, 2025).

Overall, the results of the CHC‐test and a primarily functional health literacy understanding with related activities indicate a training on critical health literacy for school nurses. The surveyed school nurses showed interest in such a training intervention.

### 4.1 Strengths and Limitations

In our study, we included various perspectives from school nurses and experts to provide a comprehensive overview of the current situation in Germany. For data collection and analysis, the various definitions and concepts of health literacy were operationalised in advance. Comparability of participating school nurses between the different investigations within this study is predominantly available, except for over‐represented academically qualified and underrepresented school nurses from international schools within the focus groups and observations. However, the study does not assess health literacy programmes in schools without an established school nurse, nor the perspectives of students and their relatives.

Only 20 participants completed the CHC‐test and it cannot be ruled out that participants made use of external aids or sources when completing the test. Reasons for the reduced sample size might be the scope or complexity of the questions; consequently, these findings are not representative. The CHC‐test results show a wide variation; reasons could lie in the small sample size or qualification‐related differences; however, subgroup analyses were not conducted due to the small sample. In addition to evidence‐based practice and critical appraisal of health information, critical health literacy also includes reflection on health determinants, empowerment, and the implementation of context specific individual or collective health measures (Abel and Benkert 2022; Benkert and Abel 2020; Steckelberg et al. 2009). The CHC‐test primarily focuses on evidence‐based aspects within the concept of critical health literacy (Steckelberg et al. 2009), in combination with the further study results, a more complex overview on critical health literacy in context of school nursing could be provided. Clarifying questions by the researcher to the anonymous, open survey answers were not possible; however, the additional qualitative investigations enabled an in‐depth analysis and complemented the results.

### 4.2 Implications

The results provide opportunities for reflection and further development of health literacy interventions, international and national. The study highlights that school nurses could meaningfully contribute to the health literacy of students and their environment within their real life. Critical health literacy is considered a relevant competence from which students could benefit. School nurses show a strong interest in the topic of critical health literacy, and to address it, and could therefore contribute significantly to it. However, the formal integration of health as a school subject and health literacy in curricula could strengthen institutional commitment and is recommended by various professional associations in Germany (Bundesärztekammer 2025). Furthermore, the interviewed experts advocate for expanding the role of school nurses as key actors in addressing health literacy in schools. The results indicate a need to increase the critical health literacy of school nurses through appropriate training programmes. In addition, providing evidence‐based materials to school nurses and ensuring the latter are trained in accordance with international standards to at least the bachelor's degree level would support them in their professional practice (Kaden and Berger‐Höger 2025b).

Further research is needed on a consistent (critical) health literacy definition, its operationalisation and appropriate objective measurement tools, as existing self‐assessments (Abel and Sommerhalder 2015; Osborne et al. 2022; Tavousi et al. 2022) are limited. There is limited research on the effectiveness of existing interventions to increase critical health literacy at schools by school nurses (in Germany) (de Buhr et al. 2020), research on this topic by involving school nurses and target groups is needed. For Germany, further research on nationwide school nursing implementation is needed.

## 5 Conclusion

School nurses aim to strengthen the health literacy of students and their environment through diverse, low‐threshold, setting‐based activities, partly within all domains of health literacy. However, existing programmes are predominantly focused on a functional health literacy understanding. The critical health literacy and evidence‐based practice of school nurses in Germany could be improved. School nurses are seen as a valuable resource for addressing health literacy in schools.

## Author Contributions

J.K.: Concept, methodology, data collection and management, data analysis, project management, evaluation, writing of the original manuscript, review and editing. L.S.: Data collection and analysis of qualitative data, review and revision of the manuscript. B.B.‐H.: Concept, methodology, supervision, review and revision of the manuscript.

## Funding

The study was self‐funded.

## Ethics Statement

Ethical approval for the study was provided by the ethics commission of the University of Bremen (file number: 2022‐17).

## Conflicts of Interest

The authors declare no conflicts of interest.

## Supporting information


**Appendix S1:** COREQ‐checklist.

**Appendix S2:** CHERRIES‐checklist.

**Appendix S3:** Category system.

## Data Availability

The data that support the findings of this study are available on request from the corresponding author. The data are not publicly available due to privacy or ethical restrictions.
