# Supplementary material for: Addressing Health Literacy by School Nurses at Schools Across Germany: Results of a Mixed‐Methods Study
Source: J Clin Nurs. 2026 Jul 31;35(12):5431–41. doi: 10.1111/jocn.70472 (PMC13744232; doi:10.1111/jocn.70472)
Supplement: Supplementary file 3 [file JOCN-35-5431-s003.pdf]

Appendix 3

Table 1 Category system with example codes „Addressing Health Literacy by School Nurses at Schools across Germany: Results of a Mixed-Methods Study" (translated from German original; HL=Health Literacy, SN=School Nurse(Nursing), S=online survey, E=expert interview, O=non-participatory observation, FG=focus group interview)

**Category/**subcategory	**Code description/definition**	**Sample code**
**Experts' perspective on health literacy/school nursing**	This code is assigned to expert statements on health literacy.	
Perception/Perspective HL/SN experts	This code is assigned to statements concerning the addressing of health literacy by school nurses from an expert perspective, for example whether it should take place, already happens or what needs exist.<br><br>Statements to interventions to increase critical health literacy are categorised to „Intervention increasing…"	"You can already set the course for this at school. And the topic Health literacy, it **means how do I get information and how can I use them for my life?** I think it´s also something that school nurses can do as, I would say, as living role model in nursing and health care. I know that if I have a problem, I can go there.<br>And then, if I have a stomach ache they´re putting a pillow on it. And I´m drinking tea and afterwards I feel better. Yes wonderful. They are good role models, like what can I actually do? And the question is, what health information can I trust? I can trust a person, who is a nurse." (E 1: 161)
Intervention increasing health literacy school (nurse)	This code is assigned to statements for ideas of intervention development to increase health literacy of school nurses, including barriers and beneficial factors.	"And by that it fits well, if you would say: Yes sure, we want to develop something or I want to develop something. And provide professionals with guidance on where and how they can pass on this skill to themselves, but perhaps also to students and teachers. And give them […] a tool. I think that´s great. I can only welcome this." (E 1: 179-181)
**School nurses' health literacy understanding**	This code contains the statements made by school nurses regarding their understanding of health literacy, according to the different dimensions of health literacy by (Nutbeam, 2000) (functional, interactive, critical), digital and professional health literacy. Statements, which don´t belong to the definitions of health literacy but for example health promotion, are assigned to the subcode discrepancies in the understanding of health literacy.	
Functional health literacy	This code is assigned when the understanding of health literacy can be matched to functional health literacy: (Nutbeam, 2000, p. 263)	"Being capable of finding and understanding and also using health informations." (S 30: 6)
Communicative/ interactive health literacy	This code is assigned to descriptions of communicative/ interactive health literacy (Nutbeam, 2000, p. 263–264).	-
Critical health literacy	This code is assigned to descriptions of critical health literacy (Nutbeam, 2000, p. 264).	"They´re also supposed to learn, where they can find trustworthy information and how to evaluate them." (S 26: 10)

Professional health literacy	This code is being used by descriptions of professional health literacy (Schaeffer et al., 2023). If it´s a description of critical handling with health information, then coding also takes place in the subcode critical health literacy.	"The knowledge of seeing and understanding health, is learned with professional experience. To effectively put knowledge into practice and learn from it." (S 46: 11)
Digital health literacy	This code is used for descriptions of digital health literacy (Kolpatzik et al., 2020). If only digital skills are described = digital health literacy, if a critical handling with digital health information are described, categorising to subcode critical health literacy.	-
Health literacy – comprehensive	This code is being used for independent decision making on health. That corresponds to the fully comprehensive definition of (Sørensen et al., 2012).	"Enabling children to make independent decisions about their health" (S 22: 6)
Discrepancies in the understanding of health literacy	This code is assigned to statements, that do not describe health literacy, but for example health promotion.	"identical definition, for students regarding learning/ for teacher refreshing/mindfulness for this topic" (S 47: 5)
Physical activity	This code is assigned to measures/ understanding movement = movement competence as a part of health literacy, cf. (Jordan et al., 2025).	"Being able to connect nutrition/physical activity and so on and health." (S 76: 7)
Nutrition	This code is assigned to measures for healthy nutrition = nutrition competence as a part of health literacy, cf. (Jordan et al., 2025).	"paying attention to a balanced diet." (S 15: 7)
Physical health	This code is assigned to measures for strengthening or addressing physical health.	"paying attention to signals from the body, such as pain, rashes, fast heartbeat, etc. - having a balanced diet - integrating exercise into daily life - knowing one's limits, [...] identifying and being aware of one's own needs (sleep, hunger, thirst, resting)" (S 15: 7)
Mental health	This code is used for strengthening mental health including conflict resolution.	"The ability of implementing measures for maintenance and/ or restoration of physical and mental health." (S 21: 8)
**Addressing critical health literacy school nurses**	This code is used for statements on how school nurses address critical health literacy.	
(Self-)assesment critical health literacy including evidence-based approach	This code is being used for the assessing of critical health literacy through school nurses or the observation of scientists. This also includes to what extent school nurses work evidence based or in what form evidence-based approach is part of their everyday life.	Topic vaccination, SN through previous positions in […] extensive knowledge/a lot of information what she can use (O 4: 34).
Participation in training critical health literacy	The code is assigned for statements regarding the extent to which SN would participate in CHL training.	Would like to participate in training for critical health literacy, if it´s offered, School nurses are lacking that (O 5: 27).
Topic critical health literacy including practice examples	This code is used for activities, that belong to the topic critical health literacy, from the perspective of SN and from the perspective of the scientist.	"I was once at a hygiene session for hand washing in a classroom, and as always... the keyword „corona" keeps coming up, and then a child started explaining that corona was caused by some Chinese people eating bats, and then I had to intervene, and that wasn't so easy, because

		teaching the children in a way that they understand that it's not right and where it comes from and so on and so forth, so yes... those were my little things“ (FG1: 80)
**Health literacy activities school**	What activities take place at your facility to promote health literacy among students? Activities are categorized in subcategories.	
No activities	This code is used for descriptions that no specific activities at school exist.	“High School: rarely prevention services – my task is to reactivate them. If specific things for increasing health literacy exist, I doubt it” (S 43: 9)
Ideas for future activities	This code is assigned, if activities are described, that haven´t yet taken place, but are planned for the future.	comprehensive health topics in every class, for example nutrition (O 4: 34)
Activities	This code is assigned, if topics or parts are described, but without description of the specific activity.	“Activities for healthy nutrition, mobility, medias and addiction, hygiene (hand washing, mouth hygiene, …), well-being” (S 34: 6)
Health groups/Working groups	This code is used, if health groups or other group offers are described, without direct relation to lessons/projects.	“Health groups for the topic nutrition, physical activity, self-perception” (S 44: 7)<br>Parents- teacher trust group (O 5: 27)
Checking vaccination records	This code is being used, if checking vaccination certificates are named as activities.	“Checking the vaccination records” (S 21: 10)
School environment	This code is assigned, if the schools environment´s design, schoolyard and so on, are described as activities	“Water dispenser: for all students water available at all times” (S 30: 8)
Services for parents	This code is used, if activities are described, that address parents. Counselling services are assigned to the category “Counselling service/training”.	“Parental involvement (parents' café)/health-related topics that parents take home” (S 78: 12)<br>“Yes, but for the children, it starts with nutrition, right? The children don't pack their lunch boxes. I also do that at parent-teacher conferences, I go to the parent-teacher conferences, grades one to five, I don't need to go to six anymore, everything's done there. But I tell them what we do in class with the children about the food pyramid according to the DGE guidelines and show them how we imagine it, how the children want it too. And if the parents then implement it, that's great, if not, well, I can't force anyone to do it, but at least we try, I try. They're still young, so that's a good place to start.” (FG2: 45)
Counselling services/training	This code is being used when parent training, counselling, or advisory services are described as an activity.	“Options of counselling services for parents, children and school staff in regard to health” (S 59: 12)
Break activities	This code is being used when activities are described that have direct reference to school breaks.	“Exercise offers at the school yard, conflict mediator (break angel?)” (S 52: 8)
Project work	This code is assigned to projects or project work that are described as activities. The projects can take place in lessons or as afternoon program.	“Projects on nutrition in grades 5 and 7 - Self-assertion in grade 8 - Method training in grade 5 - Afternoon exercise programs – school yard with climbing equipment and soccer and basketball courts - Prevention in grade 9 (drugs, smoking, alcohol)” (S 15: 9)

Offers from external partners/associations	This code is assigned when collaboration with external partners or the organization of offers from external partners is described as an activity.	"So, we address it, but I'm not competent enough in that area. When it comes to media competence, we sometimes try to get the police involved. Because sometimes the expressions and things that come up can also have legal consequences. [...] And sometimes it's also the case that the students, especially when they get a little older, need to know that too." (FG2: 140-142)
Teaching(support)	This code is assigned for teaching or co-teaching as described activity.	"Teaching units on nutrition (food science, breakfast, sugar, herbs, cooking, sugar-free muesli, learning about and tasting dairy products, cooking club) Teaching units on addiction prevention, teaching units on pulse, blood pressure, eating disorders, diabetes" (S 17: 6)
**Increasing health literacy-participation school nurses**	To what extent are they participated in these activities at schools? Question with free text from online-Survey& qualitative surveys.	
Increasing health literacy goal	This code is used when it is described that increasing health literacy is the goal of school nurses´ activities.	"Strengthen health literacy is the goal" (O 2: 37)
Participation in general	This code is assigned when the participation of SN is described, but not specifically how or in what way.	"Partially, but integration has not yet been sufficiently achieved" (S 52: 9)
No participation in activities	This code is assigned when SN´ are not actively involved in health-related topics (projects/ teaching units).	"Reluctantly, as teachers do not like to reveal their cards. Those who have not studied cannot impart health knowledge." (S 46: 14)
Acute care	This code is used for SN's description on acute care as activity.	"During treatment, I provide information about the causes and explain ways to alleviate the symptoms; show ways of prevention (e.g. fall prevention – don't leave anything on the floor). If there is pain due to injury (e.g. bruise) I explain how long it will take to recover and why moving is important despite the pain." (S 82: 9)
Idea-generator/Initiator	This code is assigned when the SN describes that she´s communicating ideas for projects/topics.	"Close cooperation with school leader in planning projects and improving [school] conditions" (S 59: 13)
Collaboration in health groups	This code is assigned when the collaboration in health- or prevention groups is described.	"Also active in teams, so, like, from bullying intervention topics to the school crisis team or protection-plan team." (FG2: 29)
Organisation	This code is assigned when SN´ describe to organise the activity. It must be checked whether she is also involved in the implementation or not.	"I´m organizing many projects from outside, for example I requested the funds for dancing projects and carried the entire organization." (S 26: 13)
Implementation with other people	This code is assigned when the activities are implemented by School Nurses together with external partners or other school staff (besides teacher). Activities that are implemented with teachers are coded in the category Implementation with teachers.	"Okay. [person4], you also got involved earlier when we were talking about cyberbullying. Or maybe_?<br>Yes, for me it's similar to the others, in that I also decided for myself, um, of course, where my priorities lie or what my knowledge is, what I can use effectively there. And where, um, I tend to go in the direction of school social work or am in contact with them. And, um, they go into classes with the topic of social learning, where the topic is also, um, what are the rules in the class chat and how do we proceed there. So those are

		also things that I pass on, so to speak, because I don't feel competent enough in that area." (FG2: 158-159)<br>„Social work does a lot for prevention, etc., and I'm only called in for that." (S 43: 10)
Implementation with teachers	This code is assigned when it´s described that activities are implemented by SN and teachers.	"I do it the way they need it now, or the nice thing is that you actually use all your skills. First, there's health literacy, which is why I'm there, but also reading and writing, which is what the teachers want. And I always find that very nice, because then the teachers can fill out the workbooks with the children and then, let's say in first or second grade, they can write themselves again. Because I can do that, I didn't learn it like a teacher did." (FG2: 243)
Independent implementation	This code is assigned when it´s described that activities are alone and independently implemented by school nurses.	„I do that as a workshop, and I also offer it on my own." (FG2: 257)
**Materials**	This code lists the (information) materials used by school nurses.	
Used materials	This code is assigned to materials that are used by school nurses as part of their work. They are described by SN or+ handed over to the researcher as part of the qualitative surveys.	„ Yes, I also like [name of project-materials]. It's been well revised, but Corona was a good opportunity for this organisation. They've worked really on differentiated material. Also, on the digital material for the children at home, on the access points. It turned out really well, but I also leave a lot out. So, you could do that, it's actually edited in such a way that you could read an entire lesson. That's what you're supposed to do, so you can do it directly if you want to. I can't do that either, I do it the way I can and understand and the way I like it. I'm just not the kind of actor who can clown around with this [used character] all the time. So I say hello once when I'm with him, and that's it, and then there are also things in between where I say, „No, I'm not doing that, I'm leaving that out." (FG2: 241)
Requirements for used materials	The code is assigned to statements regarding the extent to which specifications are made regarding the materials to be used and by whom.	"So the material [used] is, I wouldn't really call it evidence-based, but we also have a requirement from the doctor [we work together], so if we find any material on the internet, we always have to check who is the source of it? Is it some private company because they want to sell something, whatever. So we are always made aware of this, time and time again." (FG2: 273-275)
Appraisal of the used materials	This code is assigned to statements about the extent to which school nurses themselves observe quality criteria for the materials used or the importance of evidence-based practice.	what is available, checking whether official bodies such as the German Nutrition Society or BZgA, Center for Nutrition, (O 1: 12)

References

Jordan, S., Buchmann, M., Loss, J. & Okan, O. (2025). Gesundheitskompetenz und Gesundheitsverhalten – Einblicke in ein sich ausdifferenzierendes Forschungs- und Handlungsfeld für Public Health [Health literacy and health behaviour-insights into a developing field of research and action for public health]. *Bundesgesundheitsblatt - Gesundheitsforschung - Gesundheitsschutz*, *68*(3), 255–263. https://doi.org/10.1007/s00103-025-04016-6

Kolpatzik, K., Mohrmann, M. & Zeeb, H. (2020). *Digitale Gesundheitskompetenz in Deutschland.* Kolpatzik, K., Mohrmann, M. & Zeeb, H. Berlin. https://www.aok.de/pk/cl/fileadmin/user_upload/AOK-Rheinland-Hamburg/07-Presse/Dokumente/AOK_Studienbericht_Digitale_Gesundheitskompetenz.pdf. Zuletzt geprüft am 21.06.2023.

Nutbeam, D. (2000). Health literacy as a public health goal: a challenge for contemporary health education and communication strategies into the 21st century. *Health promotion international*, *15*(3), 259–267. https://doi.org/10.1093/heapro/15.3.259

Schaeffer, D., Haarmann, A. & Griese, L. (2023). *Professionelle Gesundheitskompetenz ausgewählter Gesundheitsprofessionen in Deutschland: Ergebnisse des HLS-PROF-GER*. Berlin, Bielefeld. Hertie School, Universität Bielefeld, Stiftung Gesundheitswissen.

Sørensen, K., van den Broucke, S., Fullam, J., Doyle, G., Pelikan, J., Slonska, Z. & Brand, H. (2012). Health literacy and public health: a systematic review and integration of definitions and models. *BMC Public Health*, *12*(80), 1–13. https://doi.org/10.1186/1471-2458-12-80
